# Bioassay-Guided Isolation of Anthelmintic Components from *Semen pharbitidis*, and the Mechanism of Action of Pharbitin

**DOI:** 10.3390/ijms232415739

**Published:** 2022-12-12

**Authors:** Maoxuan Liu, Jing-Guang Lu, Ming-Rong Yang, Zhi-Hong Jiang, Xiaochun Wan, Walter Luyten

**Affiliations:** 1 Center for Protein and Cell-Based Drugs, Institute of Biomedicine and Biotechnology, Shenzhen Institutes of Advanced Technology, Chinese Academy of Sciences, Shenzhen 518055, China; 2 Department of Biology, Department of Pharmaceutical and Pharmacological Sciences, KU Leuven, 3000 Leuven, Belgium; 3 State Key Laboratory of Quality Research in Chinese Medicine, Macau Institute for Applied Research in Medicine and Health, Macau University of Science and Technology, Taipa, Macau 999078, China

**Keywords:** anthelmintic activity, *Caenorhabditis elegans*, *Semen pharbitidis*, pharbitin, mechanism

## Abstract

Parasitic helminths continue to pose problems in human and veterinary medicine, as well as in agriculture. *Semen pharbitidis*, the seeds of *Pharbitis nil* (Linn.) Choisy (Convolvulaceae), is a well-known traditional Chinese medicinal botanical preparation widely used for treating intestinal parasites in China owing to its desirable efficacy. However, the anthelmintic compounds in *Semen pharbitidis* and their mechanism of action have not been investigated yet. This study aimed to identify the compounds active against helminths from *Semen pharbitidis,* and to establish the mechanism of action of these active compounds. Bioassay-guided fractionation was used to identify the anthelmintic compounds from *Semen pharbitidis*. The anthelmintic assay was performed by monitoring *Caenorhabditis elegans* (*C. elegans*) motility with a WMicrotracker instrument. Active compounds were identified by high-resolution mass spectrometry. Several (analogues of) fragments of the anthelmintic compounds were purchased and tested to explore the structure–activity relationship, and to find more potent compounds. A panel of *C. elegans* mutant strains resistant to major currently used anthelmintic drugs was used to explore the mechanism of action of the active compounds. The bioassay-guided isolation from an ethanol extract of *Semen pharbitidis* led to a group of glycosides, namely pharbitin (IC_50_: 41.0 ± 9.4 μg/mL). Hit expansion for pharbitin fragments yielded two potent analogues: 2-bromohexadecanoic acid (IC_50_: 1.6 ± 0.7 μM) and myristoleic acid (IC_50_: 35.2 ± 7.6 μM). One drug-resistant mutant ZZ37 *unc-63 (x37)* demonstrated a ~17-fold increased resistance to pharbitin compared with wild-type worms. Collectively, we provide further experimental scientific evidence to support the traditional use of *Semen pharbitidis* for the treatment of intestinal parasites. The anthelmintic activity of *Semen pharbitidis* is due to pharbitin, whose target could be UNC-63 in *C. elegans*.

## 1. Introduction

Helminth infections remain one of the most common infections in the world, especially in developing countries, with more than two billion people infected. These infections may result in devastating effects on human health, such as: anemia, stunting, developmental delays, impairment of the immune system, and worsened birth outcomes, particularly in pregnant mothers and young children [1,2]. In addition, parasitic nematodes cause dramatic economic losses in livestock and agriculture [3].

In the absence of vaccines for intestinal nematodes, the treatment of helminth infections mainly relies on chemotherapy. Despite the severe impacts caused by helminths in humans, livestock and agriculture, and their high prevalence, the arsenal of anthelmintic drugs is small [4]. In the pharmaceutical industry, the progress of anthelmintic drug discovery and development has been quite slow over the last three decades. This is partly due to the limited financial return from anthelmintic drugs and the high cost of drug development [5,6]. However, the rapid development of anthelmintic resistance has reduced the effectiveness of conventional anthelmintic drugs for the control of helminths in livestock, and occasionally in humans [7,8]. It may only be a matter of time before this phenomenon becomes common in parasites of humans [9]. Furthermore, side effects caused by current anthelmintics are significant in some cases, and have prompted usage restrictions [10]. Therefore, there is an urgent need for novel anthelmintics against parasitic nematodes [11].

Medicinal plants have been used traditionally to treat intestinal parasite infections for a long time thanks to their efficacy and safety. They can be a rich source of anthelmintic drug candidates [5,12]. *Semen pharbitidis*, the seeds of *Pharbitis nil* (Linn.) Choisy (Convolvulaceae), is a well-known traditional Chinese medicinal plant recorded in the Chinese Pharmacopeia (2020 version). The powder and the aqueous extract of the seeds are widely used in China for treating intestinal parasites owing to their desirable efficacy [13]. Additionally, it is also traditionally used as a purgative drug in China, Japan and Korea [14]. Its safety and efficacy for treating intestinal parasites has been extensively verified in humans via clinical studies [15,16,17]. The anthelmintic compounds from *Semen pharbitidis* could be promising anthelmintic hits for further drug development. Nonetheless, the anthelmintic compounds in *Semen pharbitidis* have not yet been identified.

Using parasitic nematodes as a model for anthelmintic compound screening is low-throughput, expensive and labor-intensive, because many life stages of parasitic nematodes are difficult to maintain in the laboratory. *Caenorhabditis elegans* (*C. elegans*) has been proved to be an excellent model of parasitic nematodes for anthelmintic drug discovery and characterizing the mechanism of action thanks to its similarity to parasitic species [18]. It is a free-living nematode which can be easily maintained in the laboratory and is feasible for high-throughput screening. The assessment of worm mobility is considered to be the current gold standard for measuring drug effectiveness for helminth parasites in vitro [19,20]. Thus, we chose *C. elegans* locomotion as an assay to discover novel anthelmintic compounds from *Semen pharbitidis,* and to explore their mechanism of action.

## 2. Results and Discussion

### 2.1. Bioassay-Guided Purification and Structure Elucidation of Active Compounds

**Figure 1:**
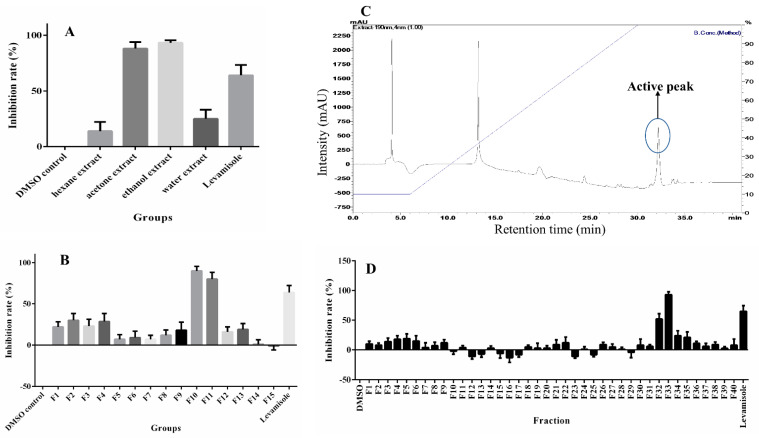
Bioassay-guided purification of active anthelmintic compounds. (**A**) Anthelmintic activity (mean ± S.D., *n* = 2) of small-scale extracts (200 μg/mL) from four different solvents. (**B**) Anthelmintic activity (mean ± S.D., *n* = 2) of fractions from a preparative silica gel column (100 μg/mL) of *Semen pharbitidis*. (**C**) HPLC chromatogram (190 nm) of pooled subfractions fr. 28–33, resolved on a C18 column at a flow rate of 1.0 mL/min. The mobile phase was a mixture of MeOH and MilliQ^®^ water (each with 0.1% TFA); the gradient is indicated by a solid blue line. (**D**) Anthelmintic activity (mean ± S.D., *n* = 2) of HPLC fractions.

Although *Semen pharbitidis* is a renowned traditional Chinese medicine used for treating helminthiasis owing to its outstanding efficacy, its anthelmintic components have not yet been identified. Therefore, bioassay-guided isolation was performed using a *C. elegans* motility assay with a WMicrotracker instrument to monitor and quantify the movement of worms. Initially, four small-scale plant extracts in different solvents were tested for anthelmintic activity. The anthelmintic results show that the ethanol and acetone extracts of *Semen pharbitidis* had significant anthelmintic activity against *C. elegans*, with the ethanol extract being the most potent among the four (Figure 1A). The potent anthelmintic activity of the crude extracts of *Semen pharbitidis* further validates its traditional anthelmintic use in China. A large-scale ethanol extract was prepared for bioassay-guided isolation of active compounds. This extract was separated by preparative silica gel column chromatography using step-wise elution to yield 15 fractions (F1–15). Two adjacent fractions, F10 and F11 (eluted by methanol (MeOH):ethyl acetate (EtOAc) (2:3 and 3:2)), had the most potent activity (Figure 1B). They were pooled and then subjected to further flash silica gel chromatography using a linear MeOH:EtOAc gradient (0–100% MeOH). The consecutive subfractions from fr. 28–33, which showed clear activity, were pooled and further separated by HPLC. Using a cutoff for the active peak of 40% inhibition, bioassay-guided isolation led to one active peak (Figure 1C,D).

**Figure 2:**
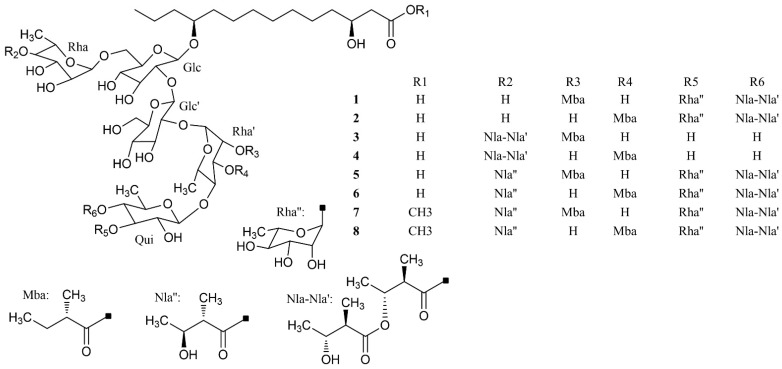
Chemical structures of identified active compounds in pharbitin isolated from *Semen pharbitidis*.

The nuclear magnetic resonance (NMR) spectrum of the active peak clearly showed the presence of saturated hydrocarbon and sugars, and indicated it to be a mixture of glycosides, instead of a pure compound (Figures S1 and S2). Although we tried many other HPLC chromatography methods (using Sephadex LH-20, NH_2_, C30, phenyl columns with different mobile phases), isolation of pure glycosides from this mixture was not successful. To identify the aglycon, we hydrolyzed the active peak with HCl under harsh conditions and analyzed the acid-hydrolyzed product with high-resolution mass spectrometry (HRMS). Two compounds were identified by extensive comparison of the HRMS results with previously published data on *Semen pharbitidis*: 11-(D-glucopyranosyloxy)-3-hydroxy-tetradecanoic acid and ipurolic acid (Figures S3 and S4). Thus, we hypothesized that the active peak was pharbitin, a mixture of oligoglycosides of ipurolic acid. Furthermore, the NMR spectrum was similar to that of pharbitin [21,22,23]. To further confirm the identity of the active peak, it was analyzed by HRMS. Eight glycosides were identified by comprehensively comparing our HRMS data with previously reported literature values of pharbitin [21,22,24] (Table S1, Figure S5d and Figure 2). Among these glycosides, compounds **7** and **8** are methyl ester derivatives of compounds **5** and **6**. To the best of our knowledge, direct isolation of the pure glycosides from pharbitin has not been achieved yet in spite of numerous attempts by natural product chemistry experts [14,21,25]. Since we could not separate the pharbitin components, we used the pharbitin for a subsequent study on its activity. Its anthelmintic potency was IC_50_: 41.0 ± 9.4 μg/mL in our assay. Given that the molecular weights of the pharbitin components range from 1306 to 1566 Da, this corresponds to a molarity of ~30 μM, compared to an IC_50_ of ~37 μM for the positive control levamisole reported in our previous study [26]. This implies that pharbitin is roughly equipotent with a major reference anthelmintic, and thus could be of great value for the development of new anthelmintics due to its relatively potent efficacy. Since pharbitin is a mixture, we cannot exclude that some of its components would have higher, and others lower IC_50_ values.

Resin glycosides from the Convolvulaceae family are well known as purgative components [25]. *Semen pharbitidis* is a main constituent of the registered drug (DA-9701) developed for treating functional dyspepsia in Korea [27]. Lately, pharbitin was demonstrated to have antibacterial and anti-seizure activity [14,24]. Remarkably, to the best of our knowledge, the anthelmintic activity of pharbitin has not yet been reported. Although pharbitin is the main active ingredient that we identified in *Semen pharbitidis*, it is still possible that other active compounds present at lower concentrations in our extract went undetected in our bioassay.

### 2.2. Anthlemintic Activity of Pharbitin Fragments and Their Analogues

**Figure 3:**
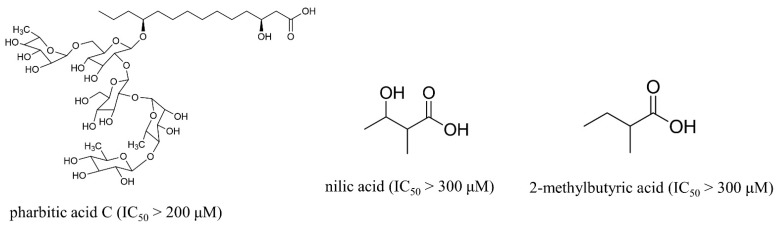
Chemical structures and anthelmintic activity of some pharbitin fragments.

Given that pharbitin is a mixture of glycosides with large molecular weights, we sought to explore the activity of its fragments. Three fragments, pharbitic acid C, nilic acid (Nla), and 2-methylbutyric acid (Mba), were tested individually, but none showed anthelmintic activity, even at high concentrations (Figure 3). This suggests that complete molecules are necessary for potent anthelmintic activity. In addition, we also performed alkaline hydrolysis for pharbitin with 1% K_2_CO_3_, as described before [24], and tested the alkaline-hydrolyzed products for anthelmintic activity, but none were active. Since the alkaline-hydrolyzed products of pharbitin are mainly pharbitic acid C, Nla, and Mba, this inactivity is consistent with the results of the three individual fragments (Figure 3).

Medium- and long-chain fatty acids have shown anthelmintic activity against *C. elegans* and other parasites [28,29]. Since pharbitin fragments were not active against *C. elegans,* and ipurolic acid, the aglycon of pharbitin, is a close analogue of myristoic acid, we performed a hit expansion for pharbitin fragments (aglycon) to search for active analogues. From a set of 20 commercially available analogues of ipurolic acid or pharbitin fragments, 2 showed potent activity: 2-bromohexadecanoic acid (IC_50_: 1.6 ± 0.7 μM) and myristoleic acid (IC_50_: 35.2 ± 7.6 μM) (Table 1). Intriguingly, neither of these compounds has not been reported to have anthelmintic activity. In addition, the activity profile of the tested analogues permitted a preliminary structure–activity relationship analysis for fatty acids. Comparing myristoleic acid, lauric acid, palmitelaidic acid with β-hydroxymyristic acid, ricinoleic acid, 12-hydroxyoctadecanoic acid, and 2-hydroxytetradecanoic acid (Table 1) suggests that the hydroxy modification on the carbon chain of the fatty acid decreases anthelmintic activity dramatically. Moreover, the inactivity of ipurolic acid, obtained by acid hydrolysis from pharbitin (Figure S2), was consistent with the preliminary structure–activity relationship analysis. Additionally, 2-bromohexadecanoic acid was the most potent compound identified in this study. Given its remarkable anthelmintic activity (IC_50_: 1.6 ± 0.7 μM), this could be a good starting point for further hit-to-lead development.

### 2.3. Mechanism of Action Study of Pharbitin

**Figure 4:**
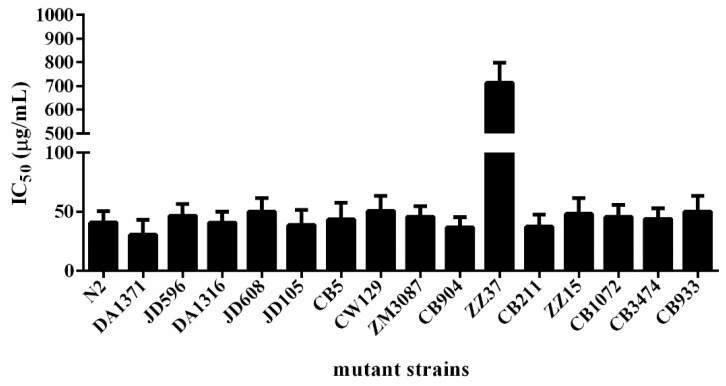
The anthelmintic activity (IC_50_) of pharbitin on drug-resistant mutant *C. elegans* strains (mean ± S.D., *n* = 3). For a list of strains, see Table 2.

Understanding the mechanism of action of anthelmintic compounds is vital for further target-based drug design to improve the compounds’ potency and selectivity. Drug-resistant mutant parasites can provide mechanistic information or evidence on whether the compound of interest targets a novel or established anthelmintic pathway [30,31]. Therefore, we tested pharbitin against a panel of *C. elegans* mutant strains (Table 2) resistant to currently used anthelmintic drugs. ZZ37 *unc-63 (x37)* demonstrated a ~17-fold increased resistance to pharbitin compared with N2 wild-type worms, while no other mutants manifested significant resistance to pharbitin (Figure 4). *Unc-63* encodes an alpha subunit of a levamisole-sensitive nicotinic acetylcholine receptor [32]. CB904, CB211, ZZ15, and CB1072 are other levamisole-resistant mutants, each with defects in different subunits of levamisole-sensitive nicotinic acetylcholine receptors (Table 2). In muscles, this receptor is composed of two non-alpha subunits, lev-1 and unc-29, and three alpha subunits unc-38, unc-63 and lev-8 [33]. Defects in any of these subunits lead to defective nicotinic acetylcholine receptors, which cannot be (sufficiently) activated any more by levamisole, leading to levamisole resistance. The ZZ37 mutant has a point mutation in *unc63* that disrupts a splice junction consensus site [32]. Although pharbitin appears to share a molecular target with levamisole in *C. elegans* (is UNC-63), its mechanism must be different. Pharbitin cannot be a receptor agonist like levamisole, nor affect upstream processes (like acetylcholinesterase), leading to altered levels of the natural agonist acetylcholine, since it is not affected by defects in other subunits. It may specifically interact directly with the UNC63 subunit, or selectively affect its synthesis or processing. Since UNC-63 is a well-established anthelmintic drug target, pharbitin has potential for development as a novel therapeutic drug for helminth diseases, although there may be cross-resistance with some levamisole mutants.

## 3. Materials and Methods

### 3.1. Chemicals and Reagents

Acetone, n-hexane, EtOAc, and MeOH, all of HPLC analytical grade, were purchased from Sigma-Aldrich (Saint Louis, MO, USA). Absolute ethanol was purchased from Fischer Chemicals (Loughborough, UK). Sterile ultra-pure water (MilliQ^®^) was produced by a water purification system (Milli-Q Reagent Water System, Merck KGaA, Darmstadt, Germany). Analogues of pharbitin fragments were purchased from Sigma-Aldrich.

### 3.2. Plant Material

Dried *Semen pharbitidis* botanical material (batch number of 20131116) was purchased from Jinan Tongrentang Pharm. Co. Ltd. (Jinan, China). The material was identified and authenticated according to the Chinese Pharmacopeia (2020 version) by local botanists.

### 3.3. Preparation of the Extract

The dried *Semen pharbitidis* botanical material was ground to a fine powder. A total of 1 g of powder was extracted in 10 mL of sterile water, absolute ethanol, hexane, or acetone. Extraction was performed at ambient temperature with the aid of repeated vortexing and sonication (4 × 15 min over a 24 h period) in a sonicator water bath (Branson, Brookfield, CT, USA). Then, 1 mL of extract was evaporated in a Savant SpeedVac Concentrator (SVC 200H, Stratech Scientific, Ely, UK), and the dried extract residue was re-dissolved in water (for the aqueous extract) or DMSO (for the ethanol, hexane, and acetone extracts) at a concentration of 40 mg/mL. For the large-scale preparation, 50 g of powdered *Semen pharbitidis* plant material was mixed with 0.5 L of ethanol, shaken and sonicated as mentioned above. The extract supernatant was evaporated under reduced pressure in a round-bottom flask using a Buchi^®^ rotary evaporator. This process was repeated twice to recover as much extract as possible. The dried extract (8.1 g) was dissolved in the round-bottom flask with a small amount of hexane, and approximately 8 g of silica gel (63–200 µm) was added, yielding a thick slurry. This mixture was then evaporated until dry.

### 3.4. Anthelmintic Activity Test

The N2 wild-type *C. elegans* strain was maintained in our laboratory and used for anthelmintic screening. Drug-resistant *C. elegans* mutant strains were purchased from the *Caenorhabditis* Genetics Center (CGC, Minneapolis, MN, USA) to study the mechanism of action (see Table 2). The worms were cultured and maintained as described earlier [26]. Briefly, synchronized populations were obtained by alkaline bleaching, after which the eggs were collected and washed with M9 buffer. Eggs in M9 were kept on a rotator at 20 °C overnight to hatch into L1s. After 24 h of incubation, L1s were transferred onto a clean NGM plate seeded with an *E. coli* OP50 lawn, and grown at 20 °C. After 48 h of incubation, young adult worms were washed from the plates with M9 buffer. The anthelmintic assay was carried out in a 96-well microplate as described earlier [26]. Briefly, freshly cultivated young adult worms were collected in M9 buffer and adjusted to approximately 3000 larvae/mL. Then, 15 μL of this suspension (containing approximately 45 larvae) was added to each well of a 96-well microplate containing 184 μL of *E. coli* OP50 culture (O.D._600_ = 0.5). Subsequently, 1 μL of test sample (stock solution or a dilution thereof in DMSO at different concentrations) was added to each well; DMSO (1 μL) was used in a separate well as a solvent control. After mixing, the 96-well microplate was placed into a WMicrotracker apparatus (Phylumtech, Santa Fe, Argentina) and incubated for 16 h at 20 °C. The movement of worms in each well was measured every 30 min and recorded by the WMicrotracker. The average number of movements over 16 h (except for the first hour) in the presence of test samples, compared with the DMSO control, was used to estimate the relative anthelmintic activity. Levamisole was used as the positive control in this assay. The same method was used to test the activity of compounds on a panel of drug-resistant *C. elegans* mutant strains (Table 2).

### 3.5. Chromatography

The dried plant material adsorbed to silica gel was evenly distributed on the surface of the bed of a silica gel column poured in hexane, and separated into 15 fractions using a step gradient consisting of solvent mixtures with progressively increasing polarity. The column was initially eluted with hexane, followed by EtOAc and hexane mixtures (EtOAc:hexane, 25:75, 50:50, 75:25, 100:0). Then, the eluting solvent mixture was changed to MeOH:EtOAc (5:95, 10:90, 15:85, 20:80, 40:60, 60:40, 100:0) and finally acetic acid:MeOH (5:95, 10:90, 15:85). Aliquots of all fractions were dried in a Savant SpeedVac Concentrator. The dried fraction materials were then dissolved in DMSO to a final concentration of 20 mg/mL, and 1 µL of this solution was used to test bioactivity against *C. elegans*. Fractions were then selected for further purification based on their activity against *C. elegans*. The cutoff for activity was set to 40% inhibition through all chromatography steps. Fractions F10 and F11 were pooled (0.91 g) and further resolved into 60 subfractions (Fr. 1–Fr. 60) by flash silica gel chromatography using a linear elution gradient of EtOAc-MeOH (0–100% MeOH). All the subfractions were tested against *C. elegans* at a concentration of 80 μg/mL. The most active subfractions (fr. 28–33) were pooled (0.36 g) and subjected to additional HPLC purification. An HPLC system (Shimadzu Corp., Kyoto, Japan) with a diode array detector was used for HPLC purification. A small aliquot of dried pooled fr. 28–33 (0.7 mg) was dissolved in 1.0 mL of 10% MeOH in water containing 0.1% trifluoroacetic acid (TFA), and purified on a C18 column (Symmetry^®^, 4.6 × 250 mm, 5 µm) using a linear MeOH gradient (10–100% over 25 min, followed by 100% MeOH for 10 min. The flow rate was 1.0 mL/min., and fractions were collected every minute. The fractions were dried in a SpeedVac Concentrator, dissolved in 12 µL of DMSO each, and tested for anthelmintic activity. The active fractions were linked to the corresponding peaks by aligning the activity profile with the corresponding chromatogram. The active peak was obtained as colorless amorphous powder with a total yield of 196.5 mg. This peak as well as its acid-hydrolyzed products (1 M HCl at 80 °C for 3 and 5 h) were analyzed by high-resolution mass spectrometry (HRMS) to obtain insight into the structure.

### 3.6. HRMS and NMR

MS analysis was performed on an Agilent 6230 time-of-flight mass spectrometer (TOF-MS). Electrospray ionization (ESI) mass spectra were acquired in positive and negative mode with ultra-pure N2 as the nebulizing and sheath gas. The results were recorded with the following ESI source parameters: capillary voltage of 3500 V, fragmentor voltage of 175 V, nebulizer of 40 psi, sheath gas flow of 11 L/min at 350 °C, and drying gas flow of 10 L/min at 325 °C. The mass analyzer was scanned from 100 to 1700 (*m*/*z*). The acid-hydrolyzed product of the active peak was hydrolyzed by 1 M HCl at 80 °C for 3 h: HR-ESI-MS *m*/*z* [M-H]^−^ calcd. 421.2443, found 421.2448, C_20_H_38_O_9_. The acid-hydrolyzed product of the active peak was hydrolyzed by 1 M HCl at 80 °C for 5 h: HR-ESI-MS *m*/*z* [M-H]^−^ calcd. 259.1915, found 259.1923, C_14_H_28_O_4_. The active peak: HR-ESI-MS *m*/*z* (1): [M-H]^−^ 1451.6936, calcd. 1451.6911, C_65_H_112_O_35_. (2): [M-H]^-^ 1305.6425, calcd. 1305.6332, C_59_H_102_O_31_. (3): [M-H]^−^ 1551.7531, calcd. 1551.7435, C_70_H_120_O_37_. (4): [M-H]^−^ 1565.7568, calcd. 1565.7592, C_71_H_122_O_37_. The HRMS data were compared with those from an earlier report [14,21,22]. NMR spectra were recorded on a 600 MHz CryoFITNMR spectrometer (Bruker, Fällanden, Switzerland) in CD_3_OD solution at the indicated temperatures. Chemical shifts are expressed in *δ* scale (ppm) using tetramethylsilane as a reference standard, and coupling constants *J* are expressed in Hz.

### 3.7. Statistical Analyses

All assays were carried out at least in duplicate and repeated at least once for confirmation. Data from dose–response experiments are represented as the percentage of inhibition, and were analyzed with GraphPad Prism 6 software (San Diego, CA, USA). A log (inhibitor) versus response non-linear fit was used to estimate the IC_50_.

## 4. Conclusions

We provide further scientific evidence to support the traditional use of *Semen pharbitidis* for the treatment of intestinal parasites. Using a *C. elegans* motility assay to isolate anthelmintic compounds from a *Semen pharbitidis* ethanol extract yielded pharbitin. Hit expansion for pharbitin fragments led to two potent analogues: 2-bromohexadecanoic acid and myristoleic acid. The study of the mechanism of action of pharbitin suggests that the anthelmintic target of pharbitin could be UNC-63 in *C. elegans*. Pharbitin is a potential novel drug candidate for helminth diseases worth investigating further.

## Figures and Tables

**Table 1:** The anthelmintic activity of (analogues of) pharbitin fragments (aglycon).

Structure	Name or Code Name	IC_50_ (μM)
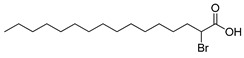	2-bromohexadecanoic acid	1.6 ± 0.7
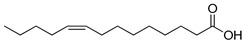	myristoleic acid	35.2 ± 7.6
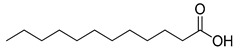	lauric acid	75.7 ± 10.3
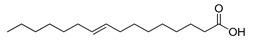	palmitelaidic acid	81.4 ± 11.7
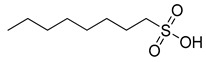	1-octanesulfonic acid	124.1 ± 21.5
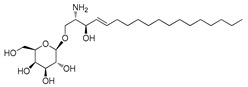	psychosine	187.6 ± 27.9
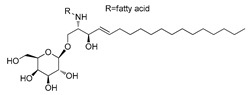	galactocerebrosides	>200
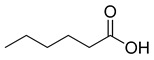	caproic acid	>200
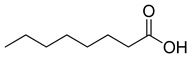	caprylic acid	>200
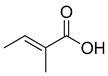	tiglic acid	>200
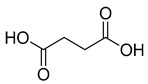	succinic acid	>200
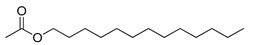	tridecyl acetate	>200
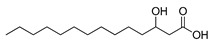	β-hydroxymyristic acid	>200
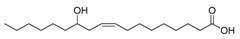	ricinoleic acid	>200
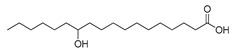	12-hydroxyoctadecanoic acid	>200
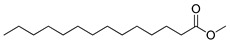	methyl myristate	>200
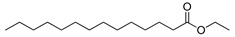	ethyl myristate	>200
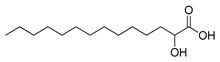	2-hydroxytetradecanoic acid	>200
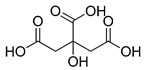	citric acid	>200
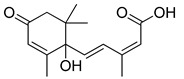	apscisic acid	>200

**Table 2:** Drug-resistant mutant strains of *C. elegans* used in this study.

Strain	Genotype	Known Resistance
DA1371	avr-14 (ad1302)	ivermectin
JD596	avr-15 (vu227)	ivermectin
DA1316	avr-14(ad1305) I; avr-15 (vu227) glc-1(pk54) V	ivermectin
JD608	avr-14(ad1305) I; avr-15 (vu227) glc-1(pk54) V	ivermectin
JD105	avr-15 (ad1051) V	ivermectin
CB5	unc-7 (e5)	ivermectin
CW129	unc-9 (fc16)	ivermectin
ZM3087	unc-9 (fc16) unc-7(e5)	ivermectin
CB904	unc-38 (e264)	levamisole
ZZ37	unc-63 (x37)	levamisole, DMPP
CB211	lev-1 (e211)	levamisole
ZZ15	lev-8 (x15)	levamisole
CB1072	unc-29 (e1072)	levamisole
CB3474	ben-1 (e1880)	benomyl
CB933	unc-17 (e245)	aldicarb

DMPP, 1,1-dimethyl-4-phenylpiperazinium.

## Supplementary Materials

The following supporting information can be downloaded at: https://www.mdpi.com/article/10.3390/ijms232415739/s1, Figure S1: ^1^H-NMR spectrum of the active peak; Figure S2: ^13^C-NMR spectrum of the active peak; Figure S3: HRMS spectra of 11-(D-glucopyranosyloxy)-3-hydroxy-tetradecanoic acid; Figure S4: HRMS spectra of ipurolic acid; Figure S5: HRMS spectra of the active peak (pharbitin); Table S1: HRMS results of pharbitin.
