# Supplementary material for: Bioassay-Guided Isolation of Anthelmintic Components from Semen pharbitidis, and the Mechanism of Action of Pharbitin
Source: Int J Mol Sci. 2022 Dec 12;23(24):15739. doi: 10.3390/ijms232415739 (PMC9779150; doi:10.3390/ijms232415739)
Supplement: Supplementary file 1 [file ijms-23-15739-s001.zip › ijms-2005940-Supplementary.pdf]

The Supplementary Materials for

Bioassay-guided isolation of anthelmintic components from *Semen Pharbitidis*, and the mechanism of action of pharbitin

Maoxuan Liu, Jing-Guang Lu, Ming-Rong Yang, Zhi-Hong Jiang, Xiaochun Wan and Walter Luyten

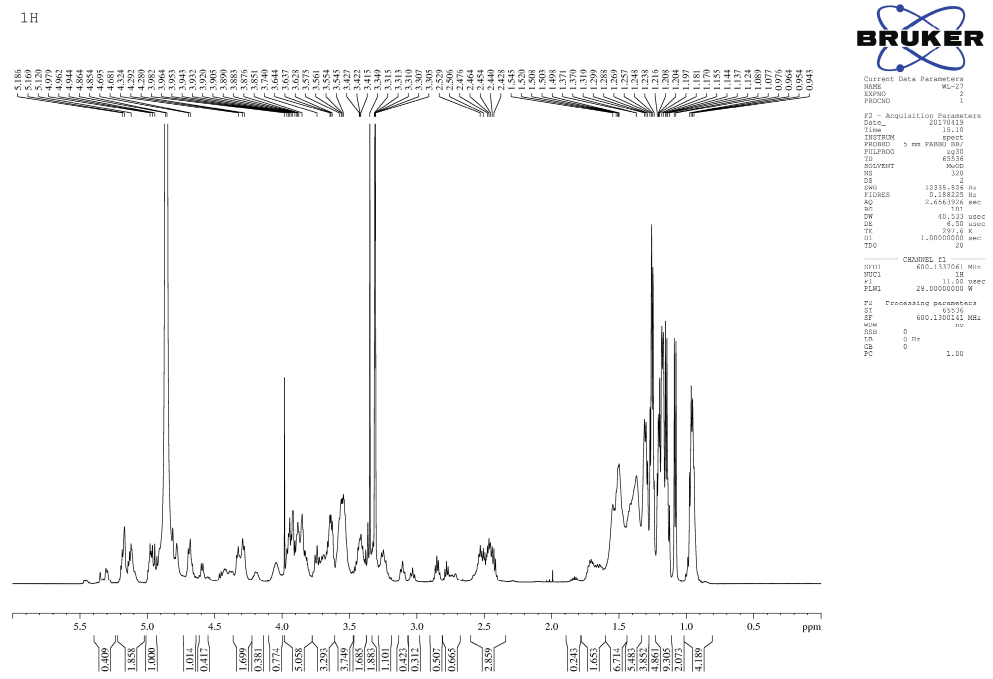
1H
5.186 5.169 5.120 4.979 4.962 4.944 4.864 4.854 4.695 4.681 4.324 4.292 4.280 3.982 3.964 3.953 3.943 3.932 3.920 3.905 3.890 3.883 3.876 3.851 3.740 3.644 3.637 3.628 3.575 3.561 3.554 3.545 3.427 3.422 3.415 3.349 3.315 3.313 3.310 3.307 3.305 2.529 2.506 2.476 2.464 2.454 2.440 2.428 1.545 1.520 1.508 1.503 1.498 1.371 1.370 1.310 1.299 1.288 1.269 1.257 1.248 1.238 1.216 1.208 1.204 1.197 1.181 1.179 1.155 1.144 1.137 1.124 1.089 1.077 0.976 0.964 0.954 0.943
5.5 5.0 4.5 4.0 3.5 3.0 2.5 2.0 1.5 1.0 0.5 ppm
0.409 1.858 1.000 1.014 0.417 1.699 0.381 0.774 5.058 3.293 3.749 1.685 1.883 1.101 0.423 0.312 0.507 0.665 2.859 0.243 1.653 6.714 5.483 3.852 4.861 9.305 2.073 4.189
BRUKER
Current Data Parameters
NAME WL-27
EXPNO 2
PROCNO 1
F2 - Acquisition Parameters
Date_ 20170419
Time 15.10
INSTRUM spect
PROBHD 5 mm PABBO BB/
PULPROG zg30
TD 65536
SOLVENT MeOD
NS 320
DS 2
SWH 12335.526 Hz
FIDRES 0.188225 Hz
AQ 2.6563926 sec
RG 101
DW 40.533 usec
DE 6.50 usec
TE 297.6 K
D1 1.00000000 sec
TD0 20
======== CHANNEL f1 ========
SFO1 600.1337061 MHz
NUC1 1H
P1 11.00 usec
PLW1 28.00000000 W
F2 Processing parameters
SI 65536
SF 600.1300141 MHz
WDW no
SSB 0
LB 0 Hz
GB 0
PC 1.00

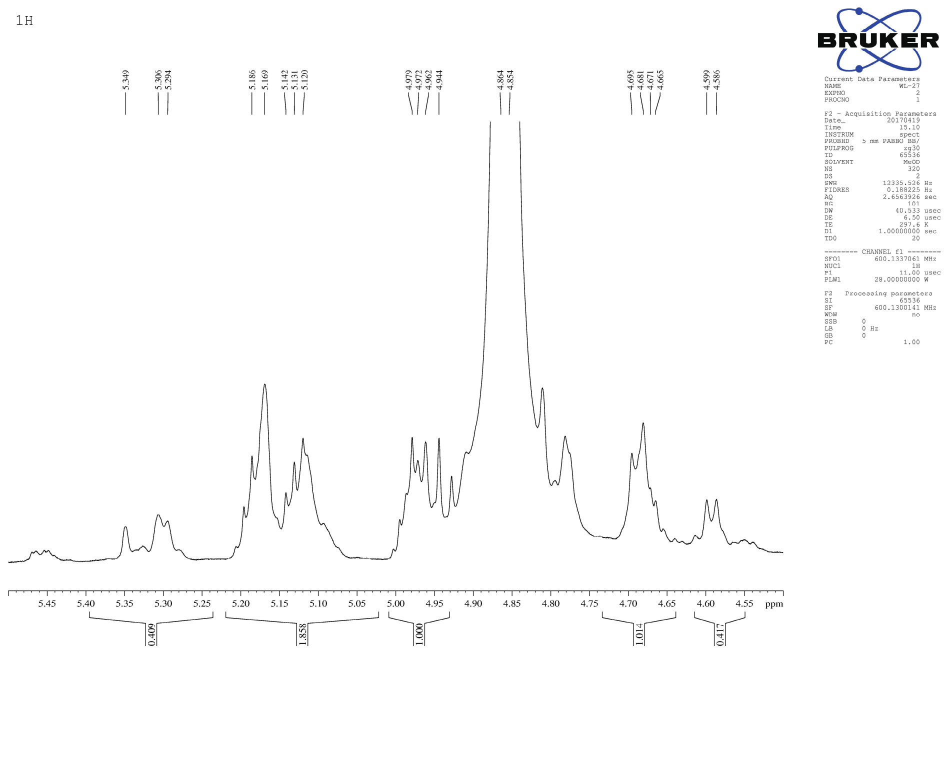
1H
5.349 5.306 5.294 5.186 5.169 5.142 5.131 5.120 4.979 4.972 4.962 4.944 4.864 4.854 4.695 4.681 4.671 4.663 4.599 4.586
5.45 5.40 5.35 5.30 5.25 5.20 5.15 5.10 5.05 5.00 4.95 4.90 4.85 4.80 4.75 4.70 4.65 4.60 4.55 ppm
0.409 1.858 1.000 1.014 0.417
BRUKER
Current Data Parameters
NAME WL-27
EXPNO 2
PROCNO 1
F2 - Acquisition Parameters
Date_ 20170419
Time 15.10
INSTRUM spect
PROBHD 5 mm PABBO BB/
PULPROG zg30
TD 65536
SOLVENT MeOD
NS 320
DS 2
SWH 12335.526 Hz
FIDRES 0.188225 Hz
AQ 2.6563926 sec
RG 101
DW 40.533 usec
DE 6.50 usec
TE 297.6 K
D1 1.00000000 sec
TD0 20
======== CHANNEL f1 ========
SFO1 600.1337061 MHz
NUC1 1H
P1 11.00 usec
PLW1 28.00000000 W
F2 Processing parameters
SI 65536
SF 600.1300141 MHz
WDW no
SSB 0
LB 0 Hz
GB 0
PC 1.00

1H

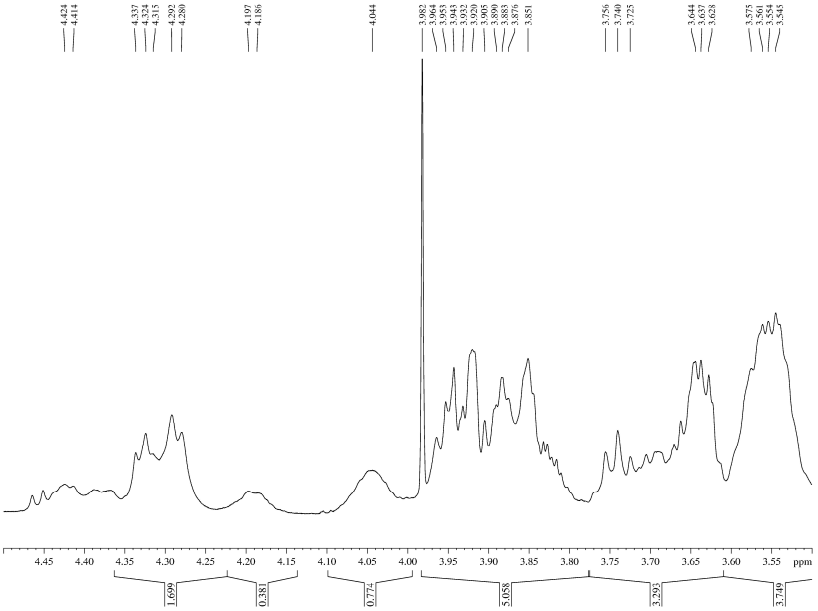

Current Data Parameters
NAME WL-27
EXPNO 2
PROCNO 1

F2 - Acquisition Parameters
Date_ 20170419
Time 15.10
INSTRUM spect
PROBHD 5 mm PABBO BB/
PULPROG zg30
TD 65536
SOLVENT MeOD
NS 320
DS 2
SWH 12335.526 Hz
FIDRES 0.188225 Hz
AQ 2.6563926 sec
RG 101
DW 40.533 usec
DE 6.50 usec
TE 297.6 K
D1 1.00000000 sec
TD0 20

======== CHANNEL f1 ========
SFO1 600.1337061 MHz
NUC1 1H
P1 11.00 usec
PLW1 28.00000000 W

F2 Processing parameters
SI 65536
SF 600.1300141 MHz
WDW no
SSB 0
LB 0 Hz
GB 0
PC 1.00

1H

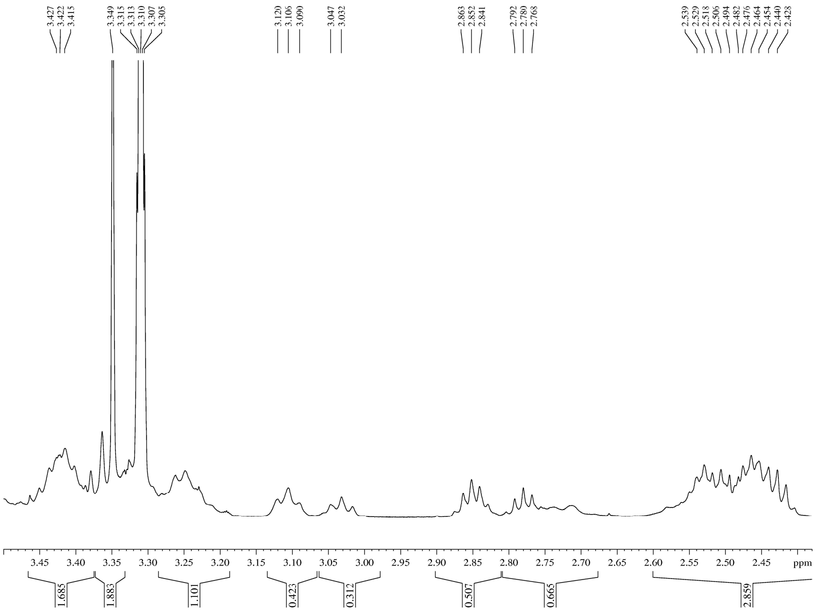

Current Data Parameters
NAME WL-27
EXPNO 2
PROCNO 1

F2 - Acquisition Parameters
Date_ 20170419
Time 15.10
INSTRUM spect
PROBHD 5 mm PABBO BB/
PULPROG zg30
TD 65536
SOLVENT MeOD
NS 320
DS 2
SWH 12335.526 Hz
FIDRES 0.188225 Hz
AQ 2.6563926 sec
RG 101
DW 40.533 usec
DE 6.50 usec
TE 297.6 K
D1 1.00000000 sec
TD0 20

======== CHANNEL f1 ========
SFO1 600.1337061 MHz
NUC1 1H
P1 11.00 usec
PLW1 28.00000000 W

F2 Processing parameters
SI 65536
SF 600.1300141 MHz
WDW no
SSB 0
LB 0 Hz
GB 0
PC 1.00

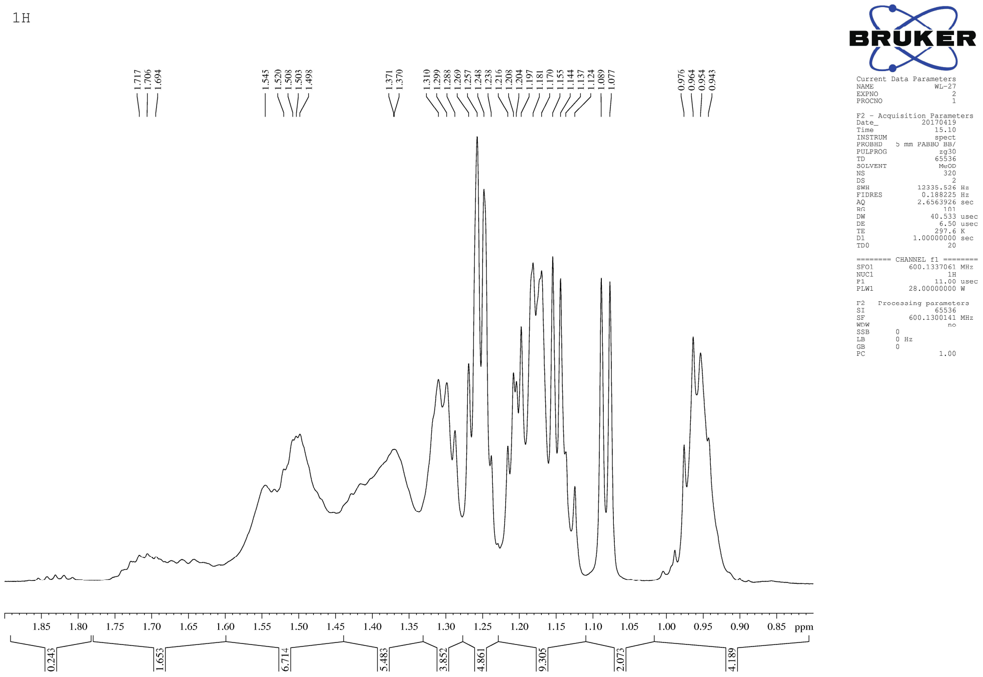

Figure. S1. ^{1}H-NMR spectrum of the active peak

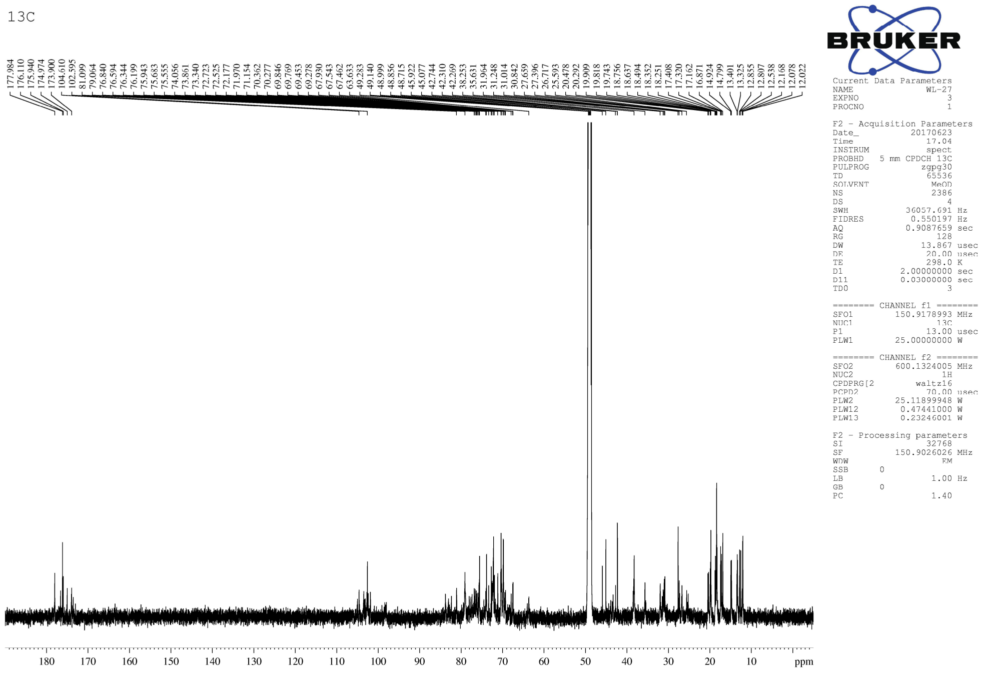

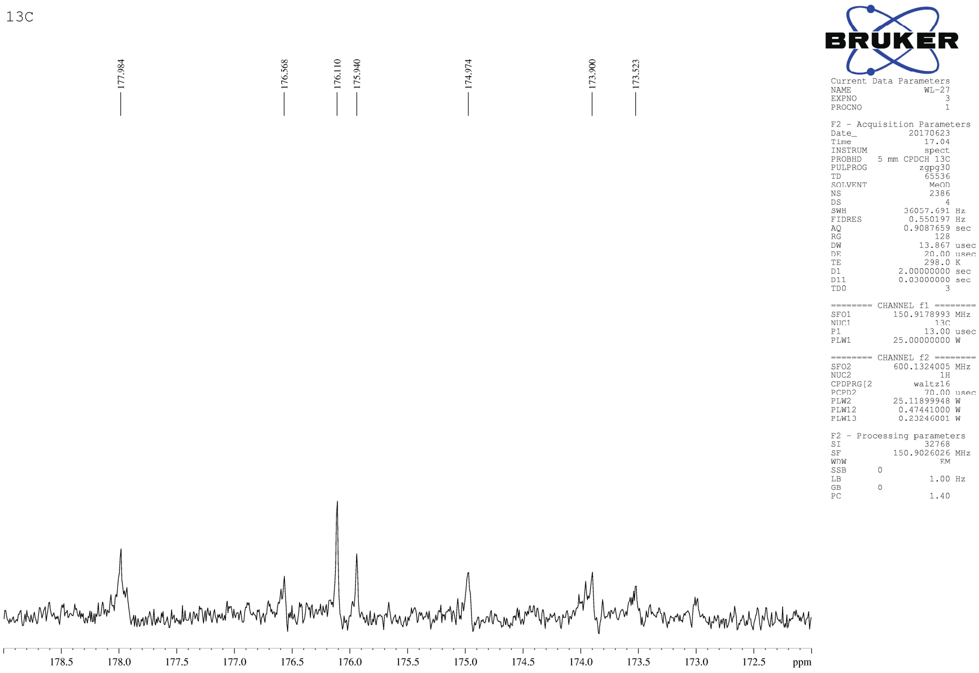
13C
177.984
176.568
176.110
175.940
174.974
173.900
173.523
178.5 178.0 177.5 177.0 176.5 176.0 175.5 175.0 174.5 174.0 173.5 173.0 172.5 ppm
BRUKER
Current Data Parameters
NAME WL-27
EXPNO 3
PROCNO 1
F2 - Acquisition Parameters
Date_ 20170623
Time 17.04
INSTRUM spect
PROBHD 5 mm CPDCH 13C
PULPROG zgpg30
TD 65536
SOLVENT MeOD
NS 2386
DS 4
SWH 36057.691 Hz
FIDRES 0.550197 Hz
AQ 0.9087659 sec
RG 128
DW 13.867 usec
DE 20.00 usec
TE 298.0 K
D1 2.00000000 sec
D11 0.03000000 sec
TD0 3
======== CHANNEL f1 ========
SFO1 150.9178993 MHz
NUC1 13C
P1 13.00 usec
PLW1 25.00000000 W
======== CHANNEL f2 ========
SFO2 600.1324005 MHz
NUC2 1H
CPDPRG[2 waltz16
PCPD2 70.00 usec
PLW2 25.11899948 W
PLW12 0.47441000 W
PLW13 0.23246001 W
F2 - Processing parameters
SI 32768
SF 150.9026026 MHz
WDW EM
SSB 0
LB 1.00 Hz
GB 0
PC 1.40

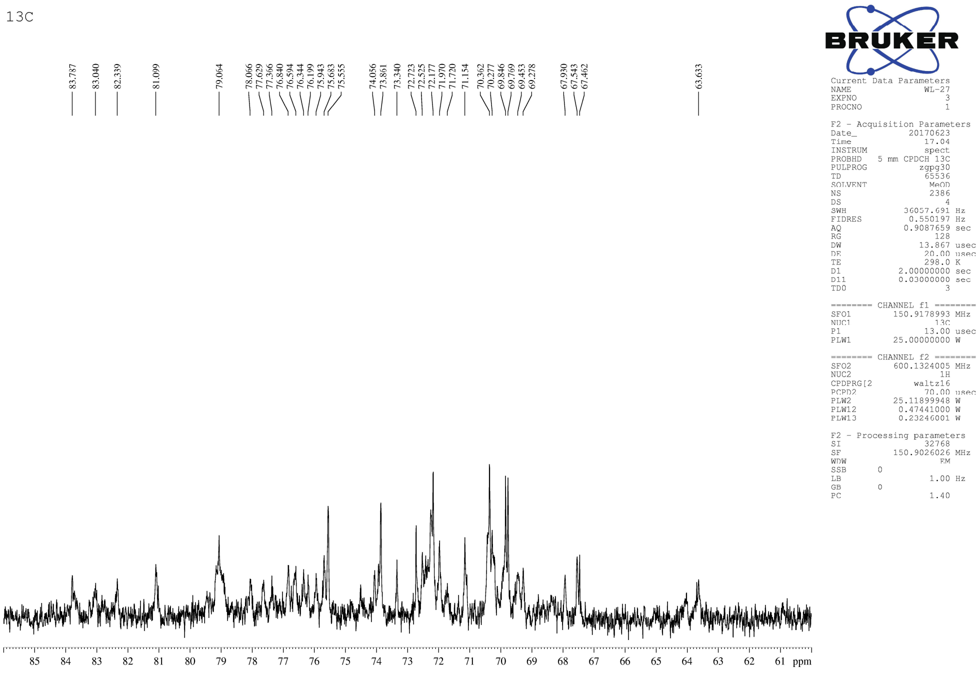
13C
83.787
83.040
82.339
81.099
79.064
78.066
77.629
77.366
76.840
76.594
76.344
76.199
75.943
75.683
75.555
74.056
73.861
73.340
72.723
72.535
72.177
71.970
71.720
71.154
70.362
70.277
69.846
69.769
69.453
69.278
67.930
67.543
67.462
63.633
85 84 83 82 81 80 79 78 77 76 75 74 73 72 71 70 69 68 67 66 65 64 63 62 61 ppm
BRUKER
Current Data Parameters
NAME WL-27
EXPNO 3
PROCNO 1
F2 - Acquisition Parameters
Date_ 20170623
Time 17.04
INSTRUM spect
PROBHD 5 mm CPDCH 13C
PULPROG zgpg30
TD 65536
SOLVENT MeOD
NS 2386
DS 4
SWH 36057.691 Hz
FIDRES 0.550197 Hz
AQ 0.9087659 sec
RG 128
DW 13.867 usec
DE 20.00 usec
TE 298.0 K
D1 2.00000000 sec
D11 0.03000000 sec
TD0 3
======== CHANNEL f1 ========
SFO1 150.9178993 MHz
NUC1 13C
P1 13.00 usec
PLW1 25.00000000 W
======== CHANNEL f2 ========
SFO2 600.1324005 MHz
NUC2 1H
CPDPRG[2 waltz16
PCPD2 70.00 usec
PLW2 25.11899948 W
PLW12 0.47441000 W
PLW13 0.23246001 W
F2 - Processing parameters
SI 32768
SF 150.9026026 MHz
WDW EM
SSB 0
LB 1.00 Hz
GB 0
PC 1.40

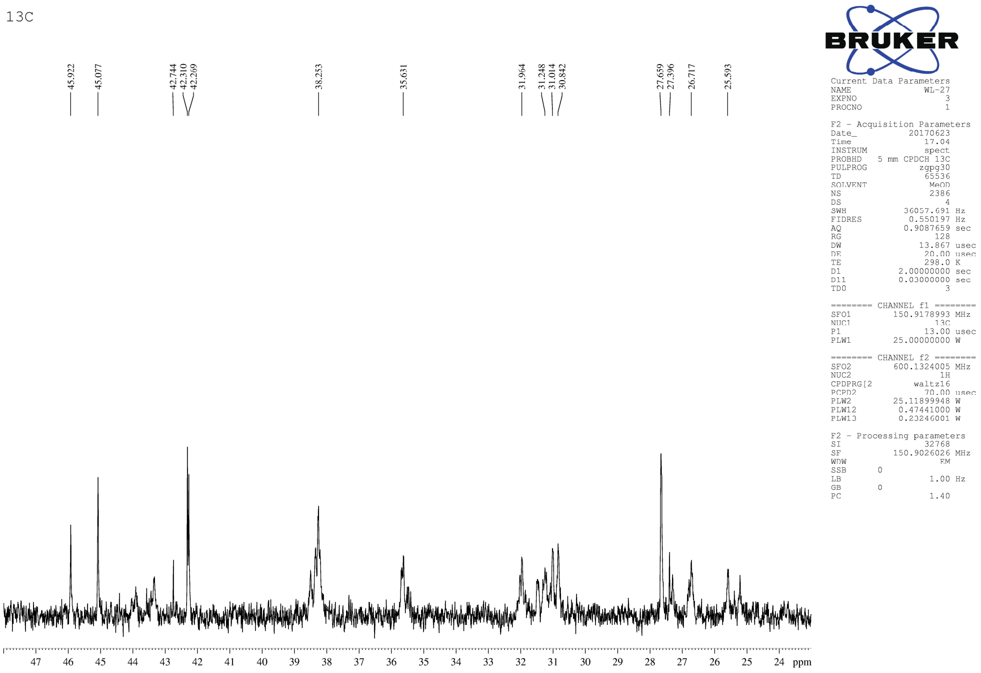

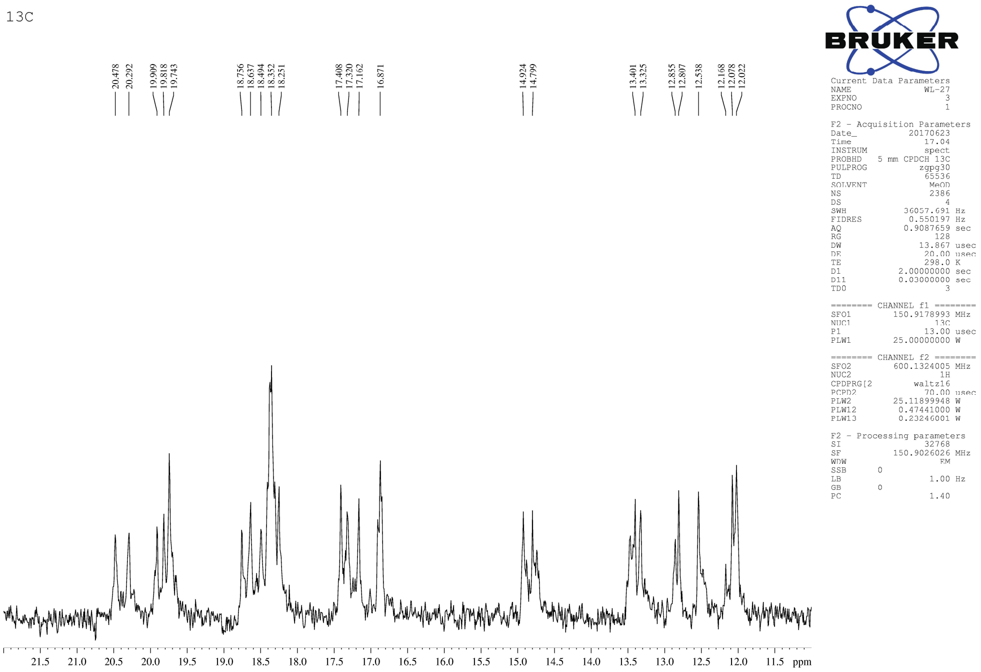

Figure. S2. ^{13}C-NMR spectrum of the active peak

11-(D-glucopyranosyloxy)-3-hydroxy-tetradecanoic acid ($C_{20}H_{38}O_9$, exact mass: 422.2516)

HRMS-ESI: $[M+H]^+$ calcd. 423.2589, found 423.2588. $[M+Na]^+$ calcd. 445.2408, found 445.2403. $[M-H]^-$ calcd. 421.2443, found 421.2448.

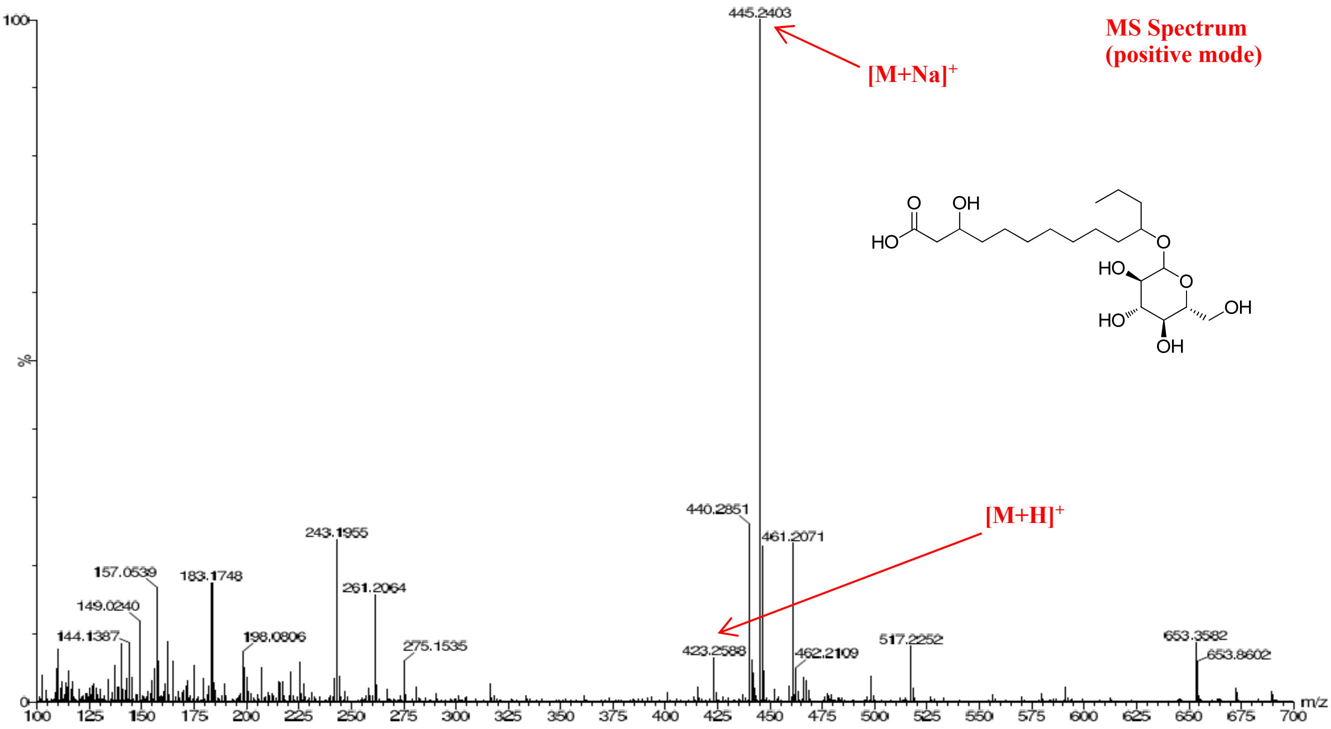

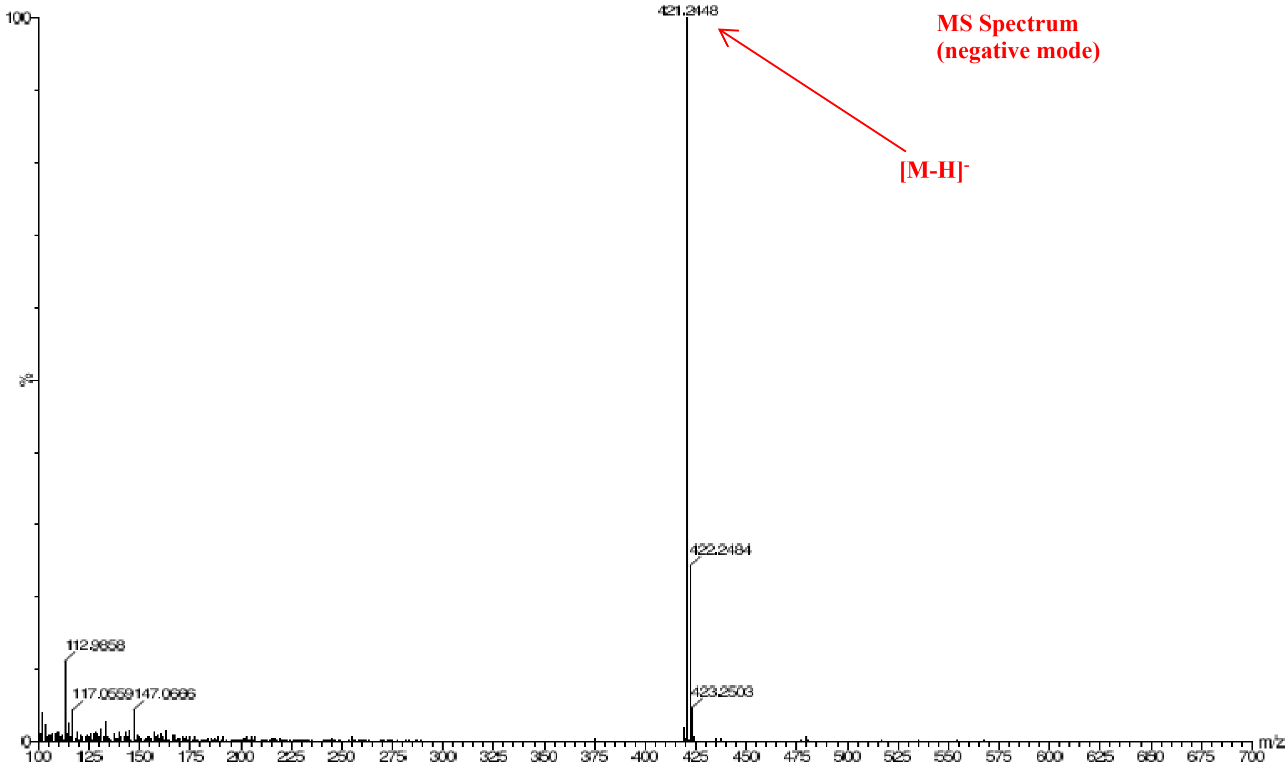

Figure. S3. HRMS spectra of 11-(D-glucopyranosyloxy)-3-hydroxy-tetradecanoic acid

Ipurolic acid ($C_{14}H_{28}O_4$, exact mass: 260.1988)

HRMS-ESI: $[M+Na]^+$ calcd. 283.1880, found 283.1877. $[M-H]^-$ calcd. 259.1915, found 259.1923.

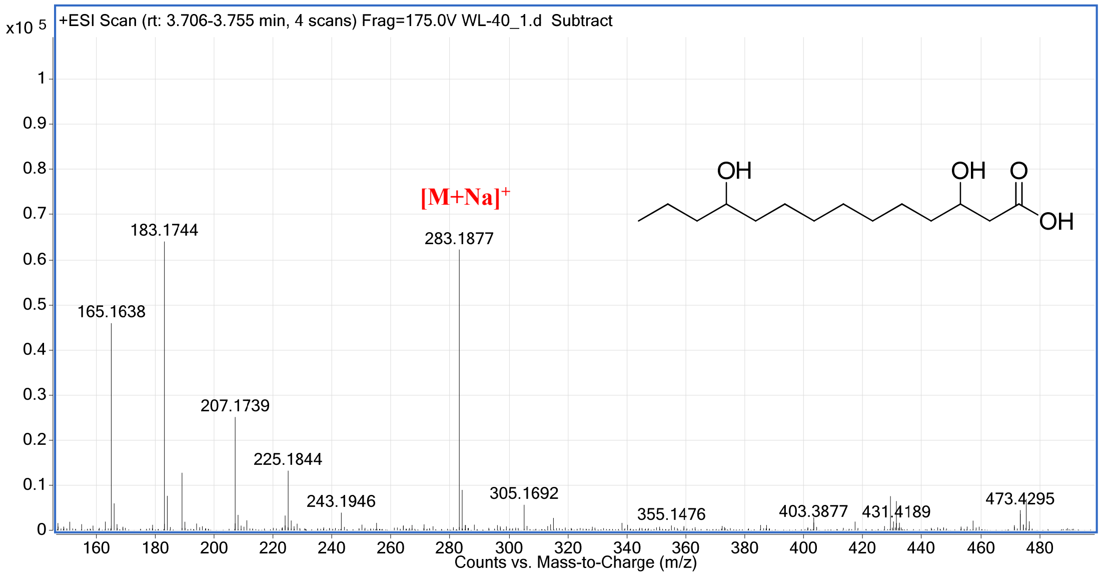

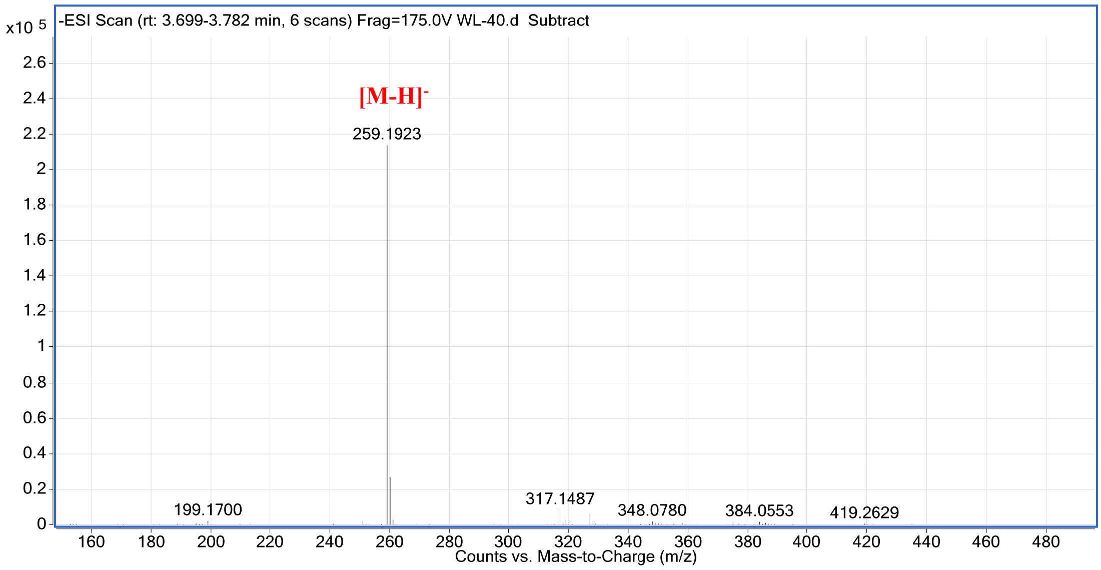

Figure. S4. HRMS spectra of ipurolic acid

Table S1. HRMS results of pharbitin

compound	formula	exact mass	calcd. [M-H]$^-$	[M-H]$^-$	calcd. [M+Na]$^+$	[M+Na]$^+$
**1**	$C_{65}H_{112}O_{35}$	1452.6984	1451.6911	1451.6936	1475.6876	1475.6933
**2**	$C_{65}H_{112}O_{35}$	1452.6984	1451.6911	1451.6936	1475.6876	1475.6933
**3**	$C_{59}H_{102}O_{31}$	1306.6405	1305.6332	1305.6425	1329.6297	1329.6827
**4**	$C_{59}H_{102}O_{31}$	1306.6405	1305.6332	1305.6425	1329.6297	1329.6827
**5**	$C_{70}H_{120}O_{37}$	1552.7508	1551.7435	1551.7531	1575.7400	1575.7380
**6**	$C_{70}H_{120}O_{37}$	1552.7508	1551.7435	1551.7531	1575.7400	1575.7380
**7**	$C_{71}H_{122}O_{37}$	1566.7665	1565.7592	1565.7568	1589.7557	1589.7495
**8**	$C_{71}H_{122}O_{37}$	1566.7665	1565.7592	1565.7568	1589.7557	1589.7495

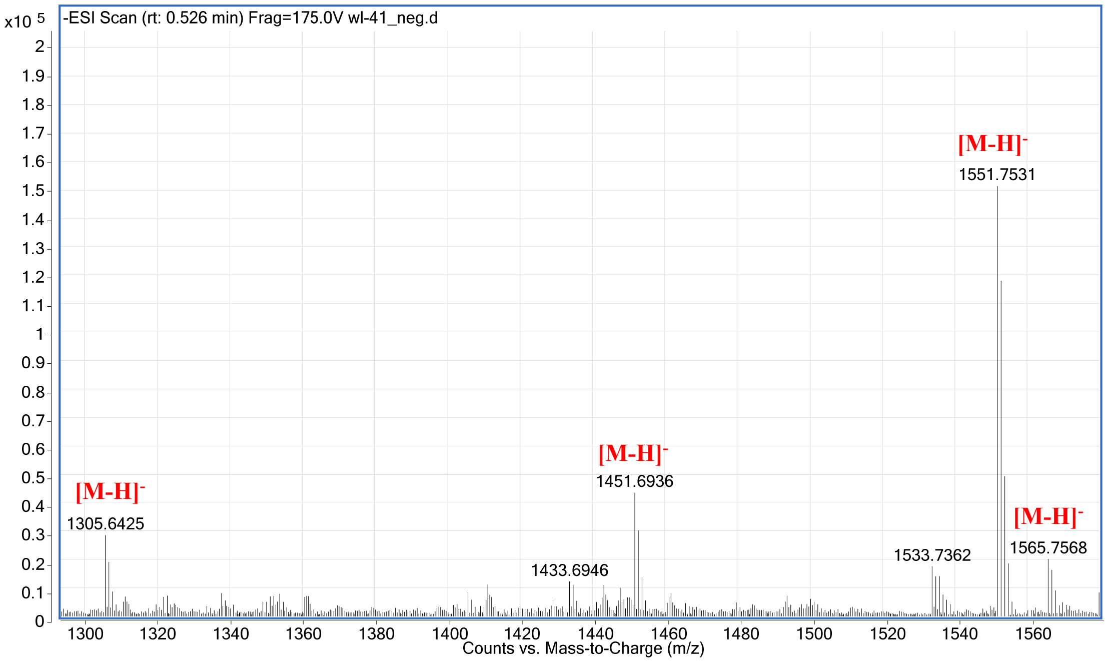

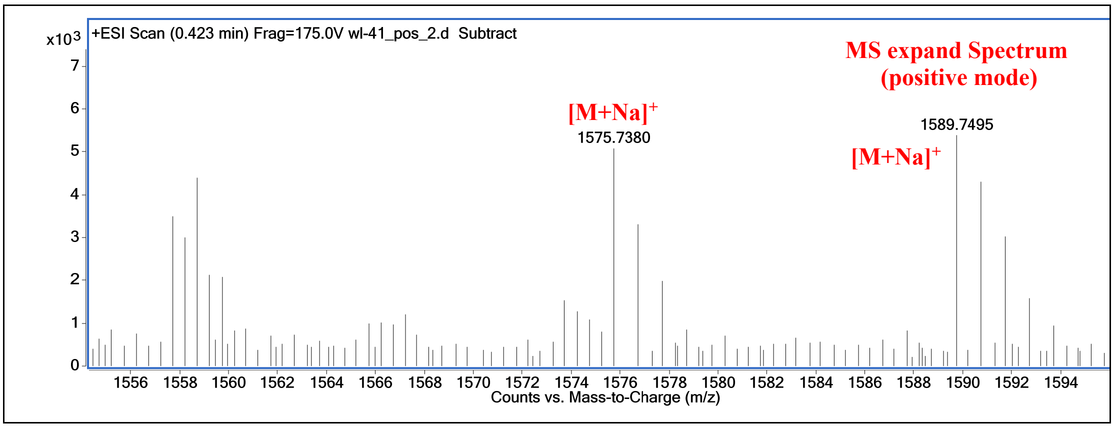

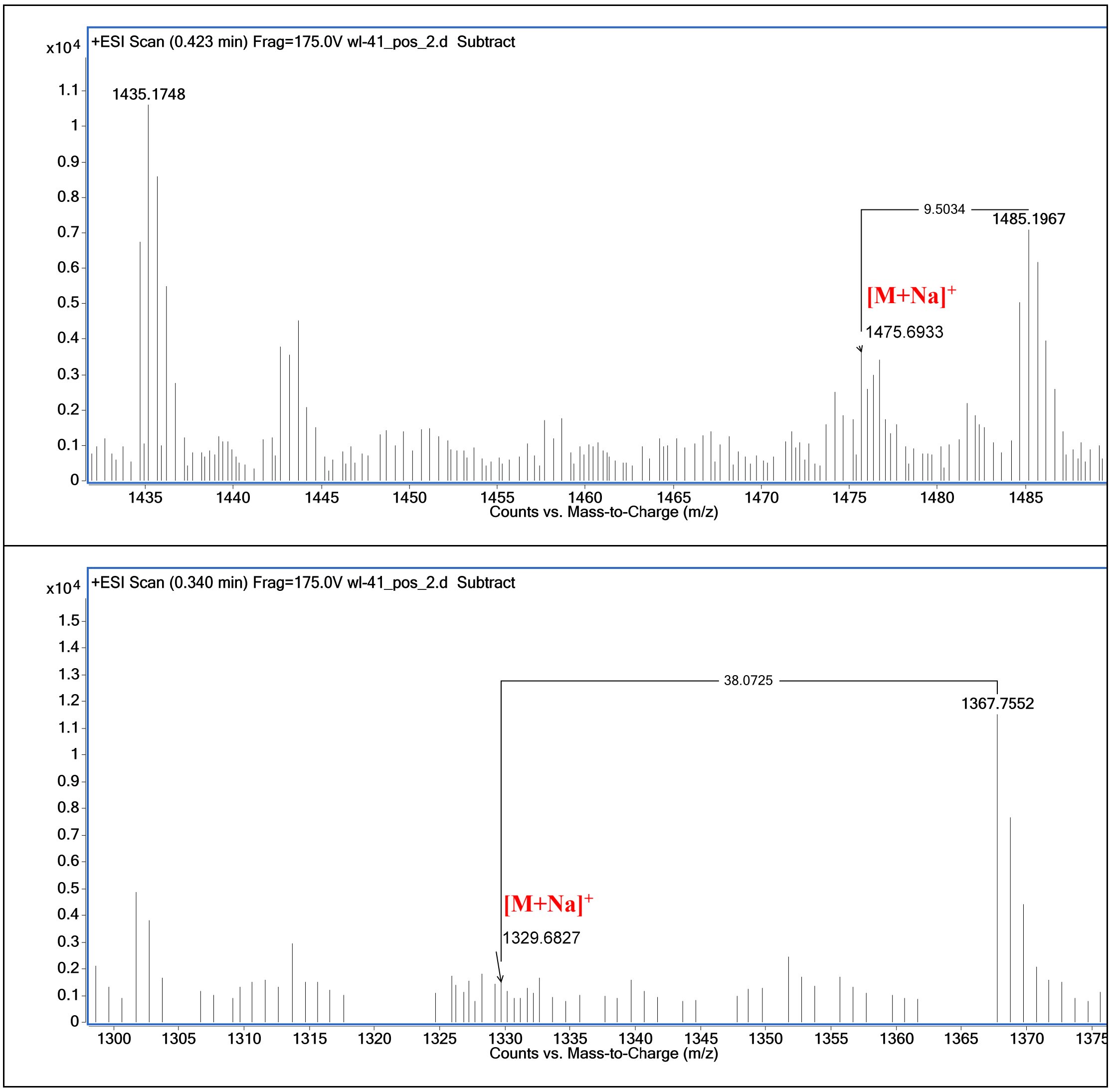

Figure. S5. HRMS spectra of the active peak (pharbitin)
